# Rapamycin in aging and disease: maximizing efficacy while minimizing side effects

**DOI:** 10.18632/oncotarget.10381

**Published:** 2016-07-01

**Authors:** Simon C. Johnson, Matt Kaeberlein

**Affiliations:** ^1^ Department of Genetics, Albert Einstein Medical College, New York, NY, USA; ^2^ Department of Pathology, University of Washington, Seattle, WA, USA

**Keywords:** mTOR, target of rapamycin, healthspan, mitochondria, mitochondria disease

## Abstract

Experimental geroscience has identified rapamycin as a top candidate for promoting healthy aging and longevity in mammals. As multiple independent studies have successfully reproduced the lifespan and healthspan promoting effects of rapamycin, the focus has shifted to possible translational use. While a promising compound, clinical use of rapamycin is limited by concerns of side effects associated with the drug. Studies aimed at defining optimal dosage regimen, delivery route, and formulation will allow for benefits to be maximized while reducing side effects.

Rapamycin is a leading candidate compound for promoting healthy longevity by targeting aging. Since 2009, more than a dozen independent studies have reported both lifespan and healthspan benefits from rapamycin treatment in mice [1]. Given these impressive results, attention has naturally shifted to potential translational applications. These include clinical trials assessing the impact of rapamycin in enhancing vaccine response in geriatric patients and on cardiac function in domestic dogs [2, 3].

Despite the promise of rapamycin (sirolimus) and related compounds (referred to as rapamycins or rapalogs), side effects are a major concern that could limit their application in clinical geroscience. Patients taking rapamycins to prevent organ transplant rejection have presented with adverse effects including stomatitis, thrombocytopenia, high serum triglycerides and cholesterol, and impaired wound healing [4]. Doses used to extend lifespan in mice are generally free from these side effects; however, lifespan-prolonging doses of rapamycin may result in a different set of adverse effects in mice, including altered glucose homeostasis (by glucose tolerance test), gonadal atrophy, and increased incidence of cataracts [5].

**Figure 1:**
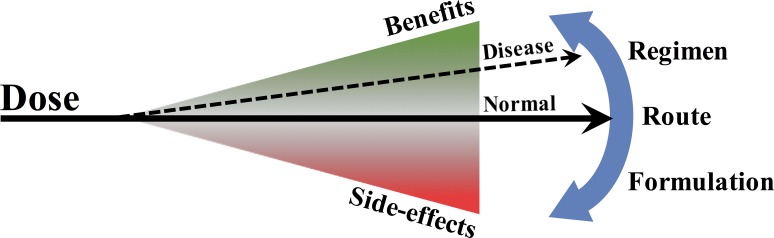
Maximizing the benefits of rapamycin Rapamycin is associated with positive outcomes and side effects which both increase with dose. Optimization of dosage regimen, drug delivery route, and formulation may provide maximum benefits while reducing off-target effects. In the setting of diseases, such as mitochondrial disease, the benefits of rapamycin may outweigh the side effects even at high dose.

Moving forward, we believe an emphasis should be placed on understanding how to achieve maximal efficacy from rapamycin treatment while minimizing side effects. In this context, limited evidence based consideration of dosing and duration of treatment represents a major limitation in most preclinical studies. The vast majority of aging studies, in particular, have utilized an encapsulated form of rapamycin (eRAPA) provided in murine chow at 14 parts per million (ppm). Only one published study has provided even a partial dose response, finding that when treatment was initiated at 9 months of age, 42 ppm eRAPA resulted in a greater magnitude of lifespan extension compared to 14 ppm, while 4.6 ppm resulted in a smaller effect [6]. Other regimens, such as administering rapamycin for two weeks each month [7] or once every five days [8], have also extended lifespan, but the relative effects among studies are difficult to interpret due to differences in strain, treatment duration, mode of delivery, and an absence of pharmacokinetic or biochemical analyses. Likewise, although short-term treatment with rapamycin can improve at least some age-associated functional measures [1], it remains unclear whether transient treatment is sufficient to recapitulate a majority of rapamycin's benefits, including longevity.

We recently set out to begin addressing the relationship between the dose of rapamycin, mode of delivery, and functional efficacy in a mouse model of severe mitochondrial disease. The *Ndufs4* knockout (KO) mouse model of Leigh syndrome is deficient in complex I of the electron transport chain and presents many features of the human disease including progressive necrotizing encephalopathy, retarded growth, lactic acidosis, and greatly reduced life expectancy. We had previously shown that daily intraperitoneal (IP) injection of rapamycin at 8 mg/kg/day dramatically attenuates disease progression and enhances survival by more than 100% [9]. More recently, we tested a range of dietary eRAPA concentrations in this mouse model, finding that lower amounts of the drug comparable to those tested for effects on lifespan in wild type mice had no effect in the KO mice [10]. Instead, much higher levels of eRAPA or IP rapamycin were needed to attenuate disease and increase survival. Based on relative phenotypic outcomes in both the KO and wild type mice, as well as serum concentrations of the drug, we conclude that dietary eRAPA must be provided at approximately 27-fold higher levels than has been commonly used (378 ppm) in order to achieve similar biological activity to daily injection of 8 mg/kg. In addition, 14 ppm eRAPA had little impact on developmental growth rate in control or diseased mice, a phenotypic readout of rapamycin activity, while higher doses robustly reduced growth. While eRAPA and injected rapamycin are not directly comparable, as the delivery methods will have different pharmacokinetic parameters and may result in dramatically different tissue distributions, these results provide an initial foray into examination of dosing and delivery of rapamycin in a preclinical model of a medically relevant class of disease.

In ongoing unpublished studies we have begun examining of the effects of transient rapamycin treatment on lifespan, healthspan, and side effects in wild type mice. These studies are being performed using both IP injection and dietary eRAPA in two different strain backgrounds. Due to resource constraints we are, by necessity, limited to a small number of potential conditions, but our hope is that these experiments will provide insight into effects of short-term rapamycin treatment, including post-treatment life expectancy and health parameters. If transient treatment recapitulates some or all of the benefits of continuous treatment, it may provide a strategy for reducing side effects while maintaining efficacy.

An additional consideration for ongoing studies are the important sex-dependent differences in the magnitude of effect of rapamycin on biological outcomes, including lifespan, with female mice generally showing a larger effect than males at lower doses of the drug. Our dose-response studies suggest that the differential impact of rapamycin on male and female mice is primarily a result of greater sensitivity of females to rapamycin, with higher doses attenuating gender differences in the phenotypic outcomes we have examined so far. Further studies, particularly at higher doses of the drug, will be required to definitively answer this question.

In summary, while remarkable beneficial impact of rapamycin on longevity and disease have now been firmly established in mice, additional studies are needed to define the relative importance of dose, delivery method, and treatment regimen. These studies are critically important for successful translation to human biology, both for understanding how to optimize beneficial effects, but equally importantly, for reducing adverse outcomes.
